# TRAPLINE: a standardized and automated pipeline for RNA sequencing data analysis, evaluation and annotation

**DOI:** 10.1186/s12859-015-0873-9

**Published:** 2016-01-06

**Authors:** Markus Wolfien, Christian Rimmbach, Ulf Schmitz, Julia Jeannine Jung, Stefan Krebs, Gustav Steinhoff, Robert David, Olaf Wolkenhauer

**Affiliations:** Department of Systems Biology and Bioinformatics, University of Rostock, 18057 Rostock, Germany; Reference und Translation Center for Cardiac Stem Cell Therapy (RTC), University of Rostock, Rostock, 18057 Germany; Gene & Stem Cell Therapy Program, Centenary Institute, 2050 Camperdown, Australia; Gene Center Munich, LMU Munich, 81377 Munich, Germany; Stellenbosch Institute of Advanced Study (STIAS), Wallenberg Research Centre at Stellenbosch University, 7602 Stellenbosch, South Africa; Sydney Medical School, University of Sydney, Sydney, NSW 2006 Australia

**Keywords:** RNA sequencing, NGS data processing, Data evaluation, Bioinformatics workflow, Galaxy, TRAPLINE, Stem cells

## Abstract

**Background:**

Technical advances in Next Generation Sequencing (NGS) provide a means to acquire deeper insights into cellular functions. The lack of standardized and automated methodologies poses a challenge for the analysis and interpretation of RNA sequencing data. We critically compare and evaluate state-of-the-art bioinformatics approaches and present a workflow that integrates the best performing data analysis, data evaluation and annotation methods in a **T**ransparent, **R**eproducible and **A**utomated **P**ipe**LINE** (TRAPLINE) for RNA sequencing data processing (suitable for Illumina, SOLiD and Solexa).

**Results:**

Comparative transcriptomics analyses with TRAPLINE result in a set of differentially expressed genes, their corresponding protein-protein interactions, splice variants, promoter activity, predicted miRNA-target interactions and files for single nucleotide polymorphism (SNP) calling. The obtained results are combined into a single file for downstream analysis such as network construction. We demonstrate the value of the proposed pipeline by characterizing the transcriptome of our recently described stem cell derived antibiotic selected cardiac bodies ('aCaBs').

**Conclusion:**

TRAPLINE supports NGS-based research by providing a workflow that requires no bioinformatics skills, decreases the processing time of the analysis and works in the cloud. The pipeline is implemented in the biomedical research platform Galaxy and is freely accessible via www.sbi.uni-rostock.de/RNAseqTRAPLINE or the specific Galaxy manual page (https://usegalaxy.org/u/mwolfien/p/trapline---manual).

**Electronic supplementary material:**

The online version of this article (doi:10.1186/s12859-015-0873-9) contains supplementary material, which is available to authorized users.

## Background

In comparison to other high-throughput methods, Next Generation Sequencing (NGS) technologies enable genome-wide investigations of various phenomena, including single-nucleotide polymorphisms, epigenetic events, copy number variants, differential expression, and alternative splicing [1]. RNA sequencing (RNAseq) uses the NGS technology for discovering novel RNA sequences, and quantifying all transcripts in a cell [2, 3]. Like genome tiling arrays, an RNAseq experiment can capture evidence for yet unannotated genes and isoforms. The utility of RNAseq to uncover new transcripts is well documented [3–8]. Several laboratories have provided evidence that cDNA library preparation and RNA sequencing sets are technically well reproducible and in contrast to microarrays RNAseq offers a broader dynamic range, which makes this platform more sensitive in the detection of transcripts with low abundance [9].

The steady increase of publications involving RNAseq experiments generated a need for statistical and computational tools to analyze the data. Basically, all RNAseq analyses involve the following tasks: pre-processing, quality control, read mapping and further analyses like differential expression (DE) analysis, single nucleotide polymorphism (SNP) analysis or gene isoform and splicing variant detection. However, the availability of tools following a standardized analysis protocol are limited [10].

A number of software packages and pipelines have already been introduced to deal with these tasks. These software packages are mainly based on programming languages like *C*, *Python* or *R* and require advanced expertise in programming or computer science for proper implementation and use or they do not provide advanced analytical tools like gene network inference methods, miRNA-target predictions and/or the integration of protein-protein interactions [11–16]. Additionally, the possibility of discovering alternatively spliced genes or promoter activity would be desirable. Furthermore, there is no common RNAseq data analysis strategy, despite the obvious need for such a standardized pipeline [17]. The increased dependence on computational approaches in life sciences has revealed grave concerns about the accessibility and reproducibility of the computed results [5]. Galaxy is a free web-based platform for omics research that addresses the following needs [18, 19]:

- *Accessibility*: Galaxy enables users to perform integrative omics analyses by providing a unified, web-based interface for obtaining omics data and applying computational tools to analyze these data. Learning a programming language or the implementation details is not necessary.
- *Reproducibility*: Galaxy produces metadata about every possible analysis step and automatically tracks descriptive information about datasets, tools, and parameter values to ensure reproducibility. User annotations and tagging is possible at each step of the pipeline.
- *Transparency*: Galaxy includes a web based framework for sharing models including datasets, histories, workflows and repositories. It also allows users to communicate and discuss their experimental results in an online forum.

## Implementation

**Fig. 1:**
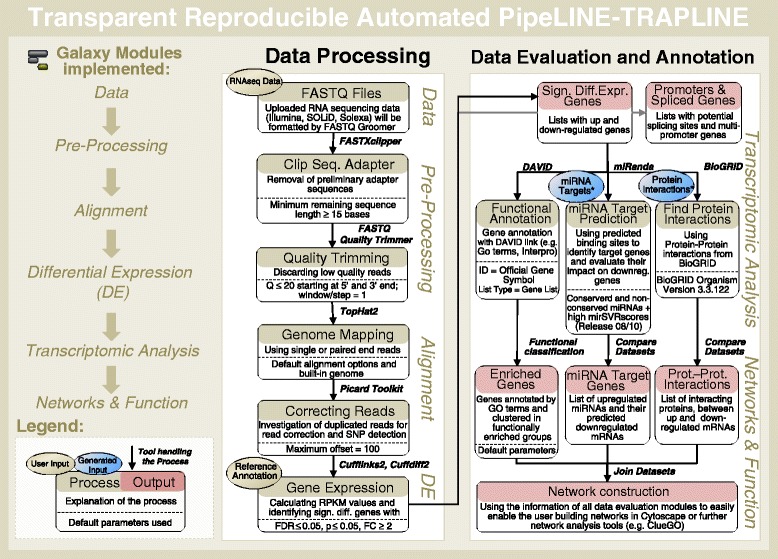
Scheme to illustrate TRAPLINE’s RNA sequencing analyses modules. * The target files are provided as Galaxy histories - *miRNA Targets* (usegalaxy.org/u/mwolfien/h/trapline-mirna-targets-input) and *Protein-Protein Interactions* (usegalaxy.org/u/mwolfien/h/trapline-protein-protein-interaction-input)

Using Galaxy, we developed a comprehensive, Transparent, Reproducible and Automated analysis PipeLINE, named TRAPLINE, for RNAseq data processing (optimized for Illumina FASTQ reads, but also suitable for other sequencing platforms like SOLiD or Solexa), evaluation and prediction. The predictions are based on modules which are able to identify protein-protein interactions, miRNA targets and alternatively splicing variants or promoter enriched sites. A schematic representation of the analysis pipeline is illustrated in Fig. 1. TRAPLINE can be accessed via the published Galaxy page of TRAPLINE (https://usegalaxy.org/u/mwolfien/p/trapline---manual) or via www.sbi.uni-rostock.de/RNAseqTRAPLINE.

TRAPLINE implements the following tools and resources: (i) FASTQ quality trimmer, FASTXclipper and FastQC for pre-processing and quality control, (ii) TopHat2 for read mapping, (iii) Picard Toolkit for read correction and SNP identification, (iv) Cufflinks2/Cuffdiff2 for DE analysis, splicing and promoter testing (v) the Database for Annotation, Visualization and Integrated Discovery (DAVID) for gene annotation and functional classification, (vi) miRanda for miRNA target prediction, (vii) BioGRID for protein-protein interactions and, finally, a compiling module for ready to use network construction files. For detailed instructions regarding the usage of TRAPLINE please see the manual in the Additional file 1.

## Results

To show the effectiveness of our automated pipeline, we exemplarily applied TRAPLINE to RNAseq data generated from our recently described antibiotic selected cardiac bodies (“aCaBs”), which are highly pure clusters of mouse embryonic stem cell (mESC) derived cardiomyocytes generated via *Myh6* promoter based antibiotic selection plus a standardized differentiation protocol (Additional file 2: Figure S1) [20, 21]. Their RNA expression profiles were compared to control embryoid bodies (EBs) derived from the same cell line without administration of the antibiotic.

### TRAPLINE includes state-of-the-art quality control processes

TRAPLINE analyses RNAseq reads obtained from Illumina, SOLiD and Solexa platforms with the help of “FASTQ Groomer” [22] that converts the specific formats as a first step. In the following pre-processing step, adapter sequences, which have been added to the 5’ and 3’ ends of the cDNA fragments during the sample preparation phase, are being clipped (no influence towards other platforms). In the Illumina sequencing procedure, sequences are extended on both ends by - 62 nucleotide long adapters that may influence the results of the subsequent analysis [23]. These adapters are only used during the Illumina bridge amplification procedure to immobilize the cDNA transcripts. In TRAPLINE we implemented the tool “FASTXClipper” (http://hannonlab.cshl.edu/fastx_toolkit/index.html) for this purpose.

It is necessary to discriminate sequencing errors from biological variation by using quality scores (Q) [24]. Therefore, in the last pre-processing step uncalled and wrongly called bases are removed (Quality Trimming). Standard approaches rely on the associated quality scores to retain the read, or a portion of it, if the score is above a predefined threshold [22]. As suggested by Mbandi et al*.* [24] we excluded reads with a score Q < 20 to ensure reliable genome mapping results. For the purpose of discarding low quality reads, we implemented the widely used tool “FASTQ Quality Trimmer” which returns a quality control report for each dataset that was analyzed. The effects achieved by quality trimming our data are shown in Additional file 3: Figure S2.

**Fig. 2:**
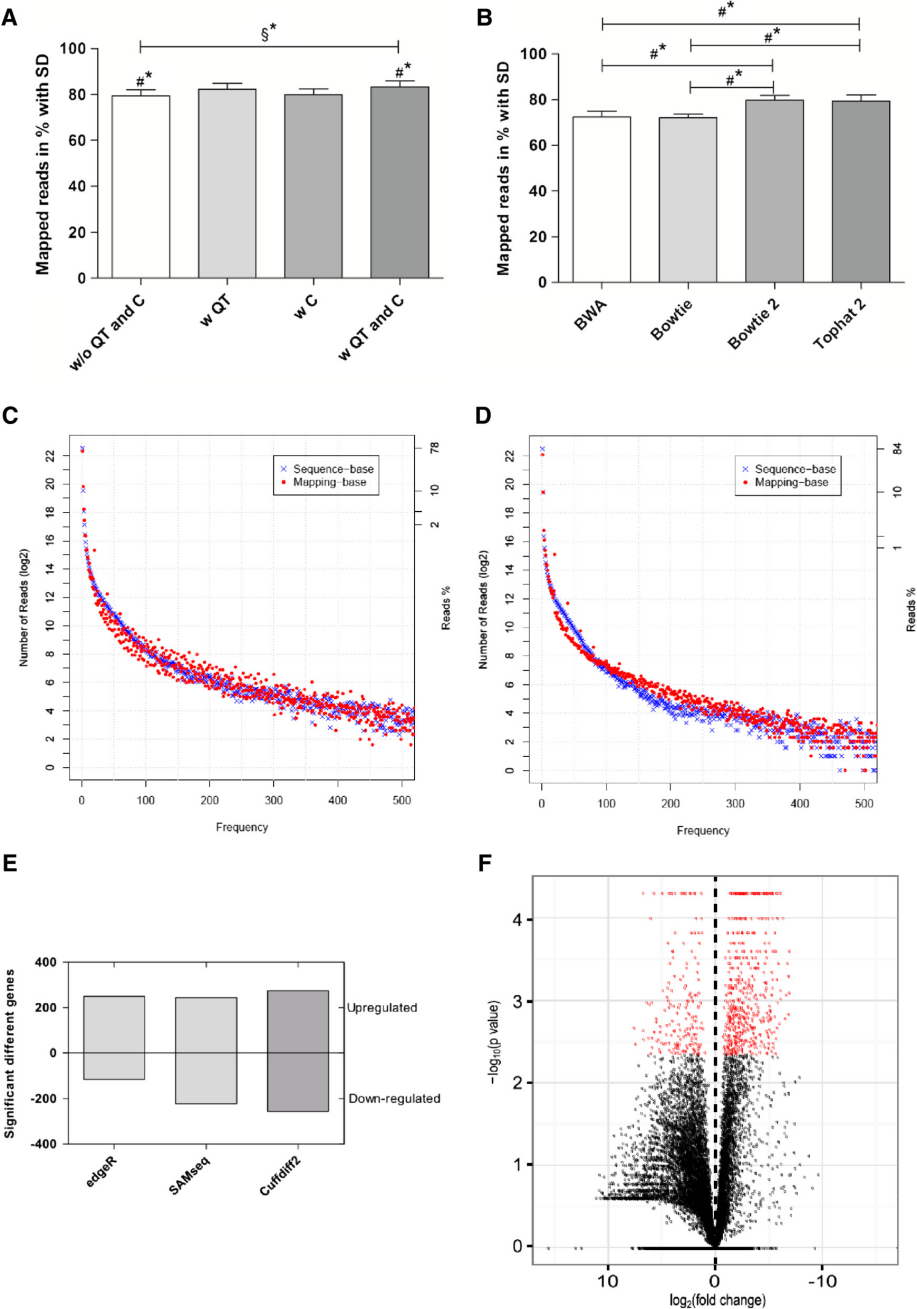
Evaluating the different analyses modules of TRAPLINE. **a** A fraction of mapped reads with and without applying pre-processing modules (QT: quality trimming; C: clipping). TopHat2 was used for genome mapping. Error bars indicate the standard deviation. Asterisks indicate a significant difference: # Welch’s *t*-test with α = 0.05; § ANOVA with α = 0.05; (*n* = 6). **b** Comparison of different genome mapping tools. The bars indicate the transcript accuracy of the reads aligned to the genome in %, including the standard deviation. Marks indicate significant difference: # Welch’s *t*-test with α = 0.05, Bonferroni test with α = 0.05; (n = 6). **c** and **d** Comparison of read correction procedure by Picard Toolkit, before (**c**) and after (**d**), to visualize and correct for multiple RNA sequences in the experimental datasets. RSeQC shows the two specific read duplication correction possibilities: "Sequence-base" reads have the same nucleotide sequence (blue), "Mapping-base" reads have the same mapped sequence, but are aligned to different locations on the genome (red). **e** Comparison of three different DE analysis tools (edgeR, SAMseq and Cuffdiff2), after read mapping with Bowtie (edgeR, SAMseq) TopHat2 (Cuffdiff2). The total number of significantly differentially expressed genes is based on FDR < 0.05 and divided into upregulated and downregulated genes. **f** Vulcano plot illustrating significantly differentially expressed genes (red dots: FC≥2; p≤0.05)

We compared the fraction of mapped reads with and without applying data pre-processing (Fig. 2a). Our observations confirm the findings of Chen et al*.* [25], who demonstrated the necessity of applying the described pre-processing steps in a read-mapping benchmark.

### TopHat2 - the most accurate alignment tool in Galaxy

To select the most suitable read alignment tool, we analyzed the overall mapped transcript coverage on the genome (accuracy) of the most commonly used alignment tools, which are based on the exon first approach. Figure 2b shows the results of our comparison between BWA [26], Bowtie [27], Bowtie2 [28], and TopHat2 [29], and their average accuracy in the mapping of six different datasets. The overall alignment accuracy of the mapped reads to the reference genome is between 70 % and 85 %. Bowtie2 and TopHat2, that share a similar algorithm, produce a significantly higher accuracy in comparison to the BWA and Bowtie alignment tools (based on a significance level α = 0.05). In our case, the Bowtie2 alignment algorithm was able to map in average 2.5 million more reads to the genome than the BWA/Bowtie algorithm (total amount reads: 24–26 million). Our observations are consistent with the results of Kim et al. [30], who found that TopHat2 generates more accurate alignments than competing tools, using fewer computational resources. Because of the significantly superior mapping accuracy of TopHat2, in contrast to Bowtie/BWA, and the additional functionality to find splice junctions and promoter regions, we decided to include TopHat2 into TRAPLINE. The outputs of TopHat2 are BAM files which contain the aligned reads to the reference genome and text files summarizing the accuracies of the mapped reads for each FASTQ file.

#### Correction of reads is necessary for SNP detection

The presence of duplicates is a major issue in single/paired short reads from NGS platforms. PCR amplification is one of the major sources of duplicates, which are usually introduced during sequencing library amplification [31]. These duplicates might have a serious impact on research applications, especially towards SNP detection, because they can confound the expression data of a particular gene and, therefore, are usually removed [32]. A popular tool for this task is “MarkDuplicates” from the Picard toolkit (https://github.com/broadinstitute/picard), which finds the 5’ coordinates and mapping orientations of each read pair and removes them. During this procedure, the tool considers all clipping that has taken place as well as any gaps or jumps in the alignment. To investigate the influence of the duplicate removing step with Picard tools, we determined the read duplication rates and the number of reads mapped to the same location using the “RSeQC” *Python* module [33]. The results are visualized in Fig. 2c and d. RSeQC uses two strategies to determine read duplication rates: (i) sequence based (blue dots), which means reads with identical sequences are regarded as duplicated reads; (ii) mapping based (red dots) which expresses reads that are mapped to exactly the same genomic location. A comparison of both figures clearly shows an elevation of the mapping-base. The red dots are refined in Fig. 2d in contrast to Fig. 2c, meaning that there were many aligned reads with a similar mapping sequence but with a different location on the genome, which was corrected by “MarkDuplicates”. The corrected bam-files can be further investigated by a SNP calling analysis software such as GATK [34] or CRISP [35].

### Cuffdiff2 adds value to the standard DE analysis

The different DE analysis methods are based on (i) negative binomial models, such as edgeR, DEseq, baySeq, (ii) non-parametric approaches, such as SAMseq, NOIseq and (iii) transcript-based detection methods, such as Cuffdiff2 and EBSeq [36]. We compared the performances of the most widely used DE tools from each group, which are Cuffdiff2 [37], edgeR [38] and SAMseq [39], to show how they compare and to underline the DE analysis efficiency of TRAPLINE. Prior to the analysis reads were mapped to the reference genome with different methods (Bowtie for edgeR/SAMseq and TopHat2 for Cuffdiff2), because each tool has different data input requirements. Figure 2e shows only slight differences between the applied DE methods. All tools nearly identified the same amount of genes as significantly upregulated among in aCaBs compared to EBs (~250), however different amounts of genes were classified as downregulated. In general, the statistical approaches used by edgeR and SAMseq are more liberal in defining significant differences than the Cuffdiff2 algorithm [17]. In agreement with our results, these widely used methods have recently been compared by several research groups [17, 36, 40, 41]. Cuffdiff2 estimates expression at transcript-level resolution and controls the variability and read mapping ambiguity by using a beta negative binomial model for fragment counts [37]. Furthermore, the tool enhances the comparability between experiments, because it uses the derived “reads per kilobase per million” (RPKM) mapped reads metric [3] which normalizes for both gene size (more reads or fragments can be mapped to larger genes) and the total number of reads or fragments (per million mapped). Seyednasrollah et al. [17] stated Cuffdiff2 as the most conservative DE method with the lowest false positive rate. Therefore, we included Cuffdiff2 for RNAseq DE analysis in TRAPLINE to retrieve precise results with highly significant genes.

As default setting Cuffdiff2 considers genes as significant for p ≤ 0.05, and a fold change (FC) higher than two. Another reason to integrate Cuffdiff2 into TRAPLINE is the possibility to determine differential splicing events and to perform differential promoter testing [42]. This possibility qualifies the pipeline to investigate for genes with two or more splice variants and genes producing two or more distinct primary transcripts (multi-promoter genes). Multiple splice and promoter isoforms are often co-expressed in a given tissue [3].

We have performed a performance test between TRAPLINE and other tools. A summary of the ratio of mapped reads, discarded reads and significantly differentially expressed genes obtained with the indicated tools is shown in Additional file 4: Table S1.

### Gene annotation, miRNA target prediction and protein-protein interactions with TRAPLINE

Additionally, we included three data annotation and prediction steps into TRAPLINE. First, filtering modules were implemented to scan the list of differentially expressed genes and extract sets of upregulated and downregulated genes. Additionally, users receive a link to DAVID [43] to evaluate the functional influences of the significantly upregulated/downregulated transcripts within their data. In general, DAVID finds Gene Ontology terms (GO terms), signaling pathways (based on databases like Panther, KEGG, Biocarta, etc.) or protein domains (e.g. based on InterPro) that are predominantly associated with lists of genes (e.g. from a DE analysis). Moreover, DAVID performs a functional annotation clustering analysis that groups these terms into functionally related clusters which gives the user a first and quick insight into the biological impact of the discovered differences [44]. Second, TRAPLINE includes modules for miRNA target prediction that use significantly upregulated and downregulated miRNAs and automatically spot possible targets among the downregulated or upregulated mRNAs in the analyzed datasets. For this purpose we provide formatted text files of conserved and non-conserved miRNAs and their predicted targets for different species (human, mouse, rat, fruitfly and nematode), based on the latest version of the microRNA.org database (*release 2010*; [45]). The files can be obtained via a Galaxy history and have to be uploaded as TRAPLINE “miRNA targets” input. Third, we implemented a module which is able to identify verified interactions between proteins of significantly upregulated and downregulated mRNAs. The protein-protein interactions are based on data from peer-reviewed publications deposited in the BioGRID database (*release 3.3.122;* [46]). Similar to the miRNA targets, we provide protein-protein interactions from five different species (human, mouse, rat, fruitfly and nematode) in the form of Galaxy history files and will continuously extend the species.

### Identifying transcriptomic differences of EBs and aCaBs

A step by step description on how to use TRAPLINE is provided in the ***Supplementary Material*** section. In summary, users upload FASTQ files from a RNAseq experiment, select the reference genome for the species under investigation and run the pipeline to obtain the significantly differentially expressed transcripts. Optionally, one can upload the provided miRNA target and protein interaction files to use the full potential of TRAPLINE. Exemplarily, we applied the developed pipeline on RNAseq data from our murine ESC derived aCaBs [21] in comparison to control EBs.

**Fig. 3:**
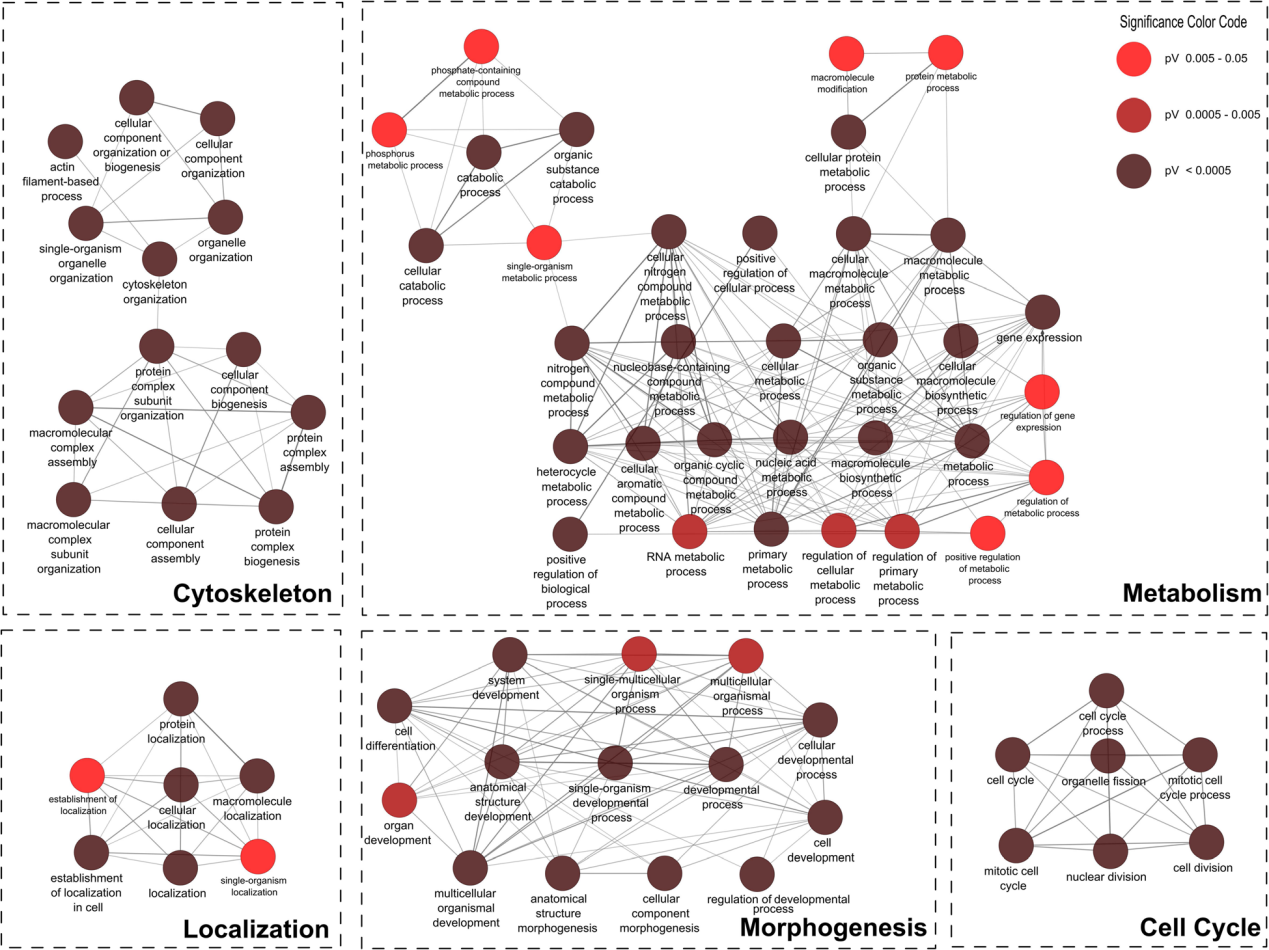
ClueGo visualization of a gene ontology interaction network obtained after functional classification analysis with DAVID. The network shows interconnections among different biological processes of 350 enriched significantly upregulated genes. Subnetworks are grouped based on GO superclasses (bold label). The colors range from red (less significant; pV = 0.05) to brown (highly significant; pV ≤ 0.05*10^−3^)

We uploaded in total six datasets (as fastqsanger files), the murine reference annotation (as gtf or gff3 that can be obtained from http://geneontology.org/page/reference-genome-annotation-project), the mm9 miRNA targets file (from the provided Galaxy history), the mm9 protein interactions file (also from the history) and ran TRAPLINE with the default parameter settings. After a processing time of ~10 h we retrieved the results. We found ~550 significantly differentially expressed transcripts, 260 of which were upregulated. The volcano plot shown in Fig. 2f illustrates the results of the DE analysis. It shows the ratio of the significantly differentially expressed genes (red) against the non-significant genes (black). At this point one might want to lower the cutoff of the p-value to obtain less reads marked as significant, which is easily possible by tuning the corresponding Cuffdiff2 parameter. However, we took the 260 upregulated genes as input for the subsequent functional classification analysis with DAVID, which revealed several annotation clusters. The first three annotation clusters contain 160 genes in total and suggest a biological impact on the cytoskeleton, actin and the contractile fibers (Additional file 5: Table S2). Based on the annotated biological processes described by GO terms, we created a network to show the links and significance of each GO term using the Cytoscape application ClueGo [47]. The network is shown in Fig. 3 and illustrates the 260 upregulated genes that are associated with enriched biological processes. The distribution of significant biological processes is illustrated in Additional file 6: Figure S3. These genes could be a starting point for subsequent analyses.

We also predicted miRNA interactions of downregulated mRNAs. Their associated GO terms suggest an impact on cardiac cell differentiation. Exemplarily, we show the significantly upregulated miRNA “mmu-mir369” with 5.522 predicted targets which include 57 genes that are downregulated in aCaBs (Additional file 7: Table S3). These 57 genes were functionally classified by DAVID and reveal a high probability to affect the cell cycle and to support cell differentiation. Among these genes is “Atp1a2” which is known to negatively regulate heart function [48]. Furthermore, we analyzed the ~550 significantly differentially expressed mRNAs and identified ~230 verified protein interactions, 10 splice variants and 12 multi promoter regions (Additional file 8: Table S4).

## Discussion

We developed TRAPLINE for RNAseq data analysis to link differentially expressed transcripts to the corresponding phenotypic changes and biological phenomena. There exist other tools such as the Bioconductor packages edgeR and DEseq [49], that are with no doubt valuable resources to support the analysis of NGS data. Our pipeline, however, includes pre-processing and genome mapping modules and, is furthermore, easily applicable. TRAPLINE mainly addresses researchers with limited or no programming skills e.g. in *R* or *Python*. We are confident that the graphical user interface of TRAPLINE, which is implemented in the Galaxy platform, greatly supports the accessibility of our RNAseq data analysis pipeline to users with no computational background. Furthermore, we have carefully selected a set of best performing interconnected modules that evade compatibility or file formatting issues. The entire RNAseq data analysis workflow can thus be performed in one go without losing the flexibility that experienced users appreciate when being enabled to adjust module parameters to their own needs. Different to other automated workflows like MeV, Chipster, RobiNA or Grape our pipeline is additionally predicting spliced variants, enriched promoter sites, miRNA targets and protein-protein interactions to enable users getting a comprehensive insight to the analyzed samples [11–14]. There are several other Galaxy pipelines available online, for example the widely used Oqtans workbench [15]. Oqtans is a collection of tools without a pre-defined pipeline. In contrast, our work for the first time introduces an automated Galaxy workflow that includes detailed data analysis and data annotation on a public Galaxy server. TRAPLINE is using all benefits of Galaxy and is independent of computational resources (*i.e.* no need for high performance computers). Researchers can access and share their data and the results worldwide via the internet, however Galaxy also offers private accounts and the possibility to install a local Galaxy instance on a private machine, which is beneficial in case of limited internet connectivity. Moreover, Galaxy enables a synchronous work, e.g. four read mapping tasks at a time are possible. In our case study the time for the analysis was reduced to 10 h in comparison to a desktop PC requiring 24 h (Additional file 9: Table S5). Additionally, to accomplish a transparent computing speed analysis, we performed a comparison between a standard TRAPLINE run at the public Galaxy instance and a local desktop PC based on a randomly selected publicly available SRA dataset (BioProject:PRJNA292442; SRA study: SRP062238) [50].

The implementation of Cuffdiff2 for detecting differentially expressed genes enhances the comparability between various RNAseq experiments, because the method is accompanied by RPKM normalization [37]. Nevertheless, it has to be considered that the RPKM value for a gene from a deep library may have more statistical meaning than an equivalent value from a more shallow library [51].

It is known that spatial biases along the genome exist, resulting in a non-uniform coverage of expressed transcripts [3]. Especially when using Cufflinks, it has been shown that DE analysis attempting to correct for differences in gene length have the tendency of introducing a bias in the per gene variances, in particular for lowly expressed genes [52]. These spatial biases hinder comparisons between genomic regions and will therefore adversely affect any analysis where such a comparison is integrated. To overcome this problem the current version of Cufflinks2 has an integrated bias correction algorithm [53]. In our investigated datasets there was no need for a bias correction, therefore, we turned this feature off (Additional file 10: Figure S4). It can be re-imported manually by setting the respective Cufflinks parameter.

With respect to the biological reliability of the results, the number of our above described 550 significantly differentially expressed genes could be further reduced based on p-value and fold change adjustments. Please be aware that the performance of our pipeline was evaluated based on the Illumina sequencing platform that was used to generate the experimental data. Additionally, it is possible to apply different multiple testing correction method like Bonferroni or Benjamini-Hochberg [54]. Using the same parameters, all three applied methods deliver similar results for differentially expressed genes. With the default parameter values, the pipeline also considers genes which are only slightly up or downregulated (|*FC*| ≥ 2). The gene annotation clustering approach enables enrichment in information and a pointer to the biological relevance of the apparently large number of differentially expressed genes. Gene Ontology terms and especially the gene set enrichment analysis performed by DAVID are established methods for gaining first insights into phenotype variations between the tested experimental conditions [43]. Interestingly, the first three enriched GO term clusters in our case study relate to biological processes concerning the cytoskeleton and actin regulation which are two core factors of cardiomyocytes and thus provide a proof of principle for our pipeline (Additional file 5: Table S2).

After successful DE analysis, there are several possibilities for further data evaluation and characterization of the transcripts. As we already showed, the GO terms and differentially expressed mRNAs can be visualized as interaction networks using Cytoscape. miRanda predictions have the largest relative overlap with other miRNA prediction algorithms/tools [55], which is why we chose to include miRanda predictions into TRAPLINE in the first place. A SNP analysis with respective tools can also be done by simply using the SNP output of TRAPLINE. Additionally, a co-expression network analysis could be performed to identify co-expressed mRNAs that are simultaneously dis-regulated [56].

## Conclusion

Taken together, our proposed pipeline includes all relevant RNA sequencing data processing modules, is easily applicable, and needs no time consuming installation processes. TRAPLINE guides researchers through the NGS data analysis process in a transparent and automated state-of-the-art pipeline. Experimentalists will be able to analyze their data on their own without learning programming skills or advanced computational knowledge. The data can be accessed worldwide and can optionally be shared among researchers. Gaining quickly in-depth insights into the biology underlying the investigated data, our work for the first time introduces an automated Galaxy workflow including detailed data processing, data evaluation and annotation modules (www.sbi.uni-rostock.de/RNAseqTRAPLINE).

## Availability and requirements

**Project name:** TRAPLINE

**Project home page:**https://usegalaxy.org/u/mwolfien/p/trapline---manual

**Operating system(s):** Platform independent

**License:** Galaxy Web Portal Service Agreement (https://usegalaxy.org/static/terms.html)

## Materials and methods

### Cell culture and aCaB-Generation

Murine ES cell lines described previously [57] were grown in high glucose DMEM with stable glutamine (GIBCO) containing 10 % FBS Superior (Biochrom), 100 μM non-essential amino acids (GIBCO), 1 % Penicillin/Streptamycin (GIBCO) and 100 μM β-Mercaptoethanol (Sigma) in presence of 1000 U/mL of Leukemia inhibitory factor (LIF, Milllipore). Differentiation of aCaBs was performed in hanging drop culture for two days using 1000 cells as starting material for one EB in Iscove’s basal medium (Biochrom) containing 10 % FBS (Biochrom), 100 μM non-essential amino acids (GIBCO), 1 % Penicillin/Streptamycin (GIBCO) and 450 μM 1-Thioglycerol. For additional 4 days, the cells were differentiated in suspension culture, and at day 6 of differentiation consistently 15 EBs were seeded on one well of a 24-well-plate. Antibiotic selection with 400 μg/mL G418 (Biochrom) was initiated at day 8 post seeding. 4 days thereafter, aCaBs were isolated via treatment with 6000 U/mL Collagenase IV (GIBCO) for 30 min. To obtain single cells for subsequent experiments, the bodies were further dissociated with 100 % Accutase (Affimetrix) for 15 min. To ensure successful generation of aCaBs, potential mycoplasma contamination was routinely controlled twice a week using the PCR based MycoSPY kit system (Biontex).

### RNA-Sequencing

For library generation and sequencing, cultured adherent cells were drained from the culture medium, washed and directly lysed by addition of lysis buffer. 1 ul of this lysate was used for cDNA Synthesis and amplification with the SMARTer kit (Clontech, Mountain View CA, USA) according to the manufacturer’s instructions. In brief, cDNA synthesis was initiated by annealing a polyA-specific primer and adding a reverse transcriptase with terminal transferase activity. The newly synthesized first strand cDNA is then tailed first with a homopolymer stretch by terminal transferase and then with a specific amplification tag by template switching. The resulting double-tagged cDNA was amplified by PCR, fragmented by sonication (Bioruptor, Diagenode, Liege Belgium; 25 cycles 30 s on/30 s off) and converted to barcoded Illumina sequencing libraries using the NEBnext Ultra DNA library preparation kit (New England Biolabs, Ipswich MA, USA). After PCR enrichment the libraries were purified with AmpureXP magnetic beads (Beckman-Coulter, Brea CA, USA) and quantified on a Bioanalyzer 2100 (Agilent, Santa Clara CA, USA). Libraries were pooled at equimolar amounts and sequenced on an Illumina GenomeAnalyzer IIx in single-read mode with a read-length of 78 nucleotides and a depth of 21 million to 32 million raw reads per replicate.

## Additional files

Additional file 1:
**TRAPLINE manual: Step by Step instructions for the usage.** (DOC 35 kb) Additional file 2: Figure S1. Flowchart for aCaB Generation. Cartoon is displaying sequential steps for the generation of aCaBs, combining *Myh6*-promoter selection and an additional cell-dissociation step [21]. (TIF 90 kb) Additional file 3: Figure S2. Visualization for RNA transcript quality control and comparison of per base quality score Q. The images are taken before (A) and after (B) quality trimming procedure (removes reads with Q ≤ 20) to estimate the effect of trimming. The quality score Q is plotted to the read position by using the FastQC package in Galaxy (*http://www.bioinformatics.babraham.ac.uk/projects/fastqc/*). The color indicates the quality of the read: "red" low quality, "orange" median quality, "green" good quality. Red line expresses the mean of the measured values (yellow boxes are inter-quartile range) and the blue line represents the mean quality. (ZIP 81 kb) Additional file 4: Table S1. Performance comparison of TRAPLINE *vs* other tools. (DOC 28 kb) Additional file 5: Table S2. Example for DAVID functional gene annotation clustering of significantly differentially expressed genes from aCaBs and EBs. (DOC 48 kb) Additional file 6: Table S3. Exemplarily we show a result of a miRNA target prediction analysis of TRAPLINE. (TIF 84 kb) Additional file 7: Figure S3. Pie chart illustrating enriched biological processes of upregulated genes in the aCaB derived cardiomyocytes. The chart presents the enriched GO superclasses. (DOC 26 kb) Additional file 8: Table S4. Exemplarily results of protein-protein interaction prediction, splice variants and multi promoter regions. (DOC 37 kb) Additional file 9: Table S5. Benchmarking results of TRAPLINE performed on a public Galaxy server and on a local desktop PC (based on computing speed). (DOC 31 kb) Additional file 10: Figure S4. A comparison of experiments without (A) and with (B) bias correction performed with the help of Cufflinks2. The dots represent the dependency of the log ratio of two FPKM values (M) and their mean average (A). The MA plot is a common method to investigate the biases of datasets [53]. (PPTX 236 kb)
