# Supplementary material for: TRAPLINE: a standardized and automated pipeline for RNA sequencing data analysis, evaluation and annotation
Source: BMC Bioinformatics. 2016 Jan 6;17:21. doi: 10.1186/s12859-015-0873-9 (PMC4702420; doi:10.1186/s12859-015-0873-9)
Supplement: Additional file 1: — TRAPLINE manual: Step by Step instructions for the usage. (DOC 35 kb) [file 12859_2015_873_MOESM1_ESM.doc]

# TRAPLINE manual: Step by Step instructions for the usage

- - **Do** your experiments (Illumina Sequencing) and obtain the **FASTQ files** (Illumina, SOLiD, Solexa),
    - **Note**: the analysis is predefined for the comparison of two experimental conditions with a triplicate for each experimental setup
  - **Go** to the Galaxy website [https://usegalaxy.org](https://usegalaxy.org/)
  - If you are new to Galaxy please **create** an account
  - **Import** our developed analysis workflow **TRAPLINE** through [www.sbi.uni-rostock.de/RNAseqTRAPLINE](http://www.sbi.uni-rostock.de/RNAseqTRAPLINE) or use the [Galaxy page “TRAPLINE”](https://usegalaxy.org/u/mwolfien/p/trapline---manual) (Use the small green “+” icon for an import)
  - (Optional): Edit the settings or parameters, especially if you want to use less replicates than 3 please adjust the workflow
  - **Upload** your FASTQ datasets (6 slots are predefined, 2 conditions with 3 replicates per condition)
    - **Choose** format “*fastqsanger*” for uploading your data (use the “*Get data”* icon on the left site)
      - You have two possibilities for uploading your data:
        - Direct upload from your hard drive
        - Upload data from a FTP server
  - **Upload** a reference annotation set for your species as a .gtf file (here: mm9) and assign it to the “*Reference annotation*” input file of the workflow.
    - The latest version of your specific species can be obtained via <http://geneontology.org/page/reference-genome-annotation-project> as gtf annotation file
  - (Optional): **Upload** a miRNA target file from the Galaxy history “[miRNA Targets](https://usegalaxy.org/u/mwolfien/h/trapline-mirna-targets-input)” for your species of interest and assign it to the “*miRNA target prediction*” input file of the workflow.
    - We provide formatted ready to use miRNA target prediction files for human, mice, rat, fruitfly and nematode based on [microRNA.org](http://www.microrna.org/microrna/getDownloads.do).
  - (Optional): **Upload** a protein interaction file from the Galaxy history “[Protein Interactions](https://usegalaxy.org/u/mwolfien/h/trapline-protein-protein-interaction-input)” for your species of interest and assign it to the “*Protein interaction*” input file of the workflow.
    - We provide several formatted and ready to use protein-protein interaction files based on [BioGRID](http://thebiogrid.org/).
  - **Go** to the “*Workflow*” section, select “RNASeqTRAPLINE” and click on Run (Please be aware that your maximum Galaxy storage space is 250 GB, therefore check your remaining space before each run)
  - **Assign** your six datasets to the given order (have a look at the annotation text) and choose your reference annotation file
  - **Assign** the type of your RNA sequencing FASTQ files within the FASTQ Groomer modules (Illumina, SOLiD or Solexa)
  - **Select** a reference genome of species for each TopHat2 alignment as a *Galaxy build-in* (mice mm9 is predefined)
    - We used the default TopHat2 parameter adjustments as recommended by Kim *et al.* [30].
    - The single end read mode is also predefined, but can be changed in the TopHat2 settings
    - Moreover, Trapnell *et al.* [29] recommended to avoid the use of genome reference annotation in the genome alignment step, because this step would prevent the identification of novel, yet uncharacterized, transcripts.
  - **Start** the workflow
  - **Obtain** your results
    - A list of all genes and additional a list containing only the significantly differentially expressed genes
    - A list of differential splice variants of each primary transcript
    - A list of differential promoter use between the samples
    - A list of significant upregulated / downregulated genes
    - Link to DAVID to further analyze the obtained significantly differentially expressed genes regarding their annotation and impact to the phenotype (Please rerun the module with column 3 as identifier)
    - A read corrected .bam file for SNP analysis
    - A list of significantly up regulated / down regulated miRNAs including their predicted targets that are also significantly up regulated / down regulated
    - A list of protein-protein interactions based on up regulated mRNAs
    - A ready-to-use list to start importing your obtained data into Cytoscape for network analysis
