# Supplementary figures and images for: TRAPLINE: a standardized and automated pipeline for RNA sequencing data analysis, evaluation and annotation
Source: BMC Bioinformatics. 2016 Jan 6;17:21. doi: 10.1186/s12859-015-0873-9 (PMC4702420; doi:10.1186/s12859-015-0873-9)

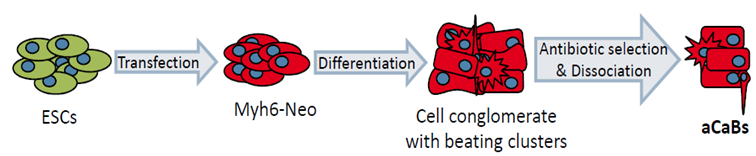

Supplement: Additional file 2: Figure S1. — Flowchart for aCaB Generation. Cartoon is displaying sequential steps for the generation of aCaBs, combining Myh6-promoter selection and an additional cell-dissociation step [21]. (TIF 90 kb) [file 12859_2015_873_MOESM2_ESM.tif]

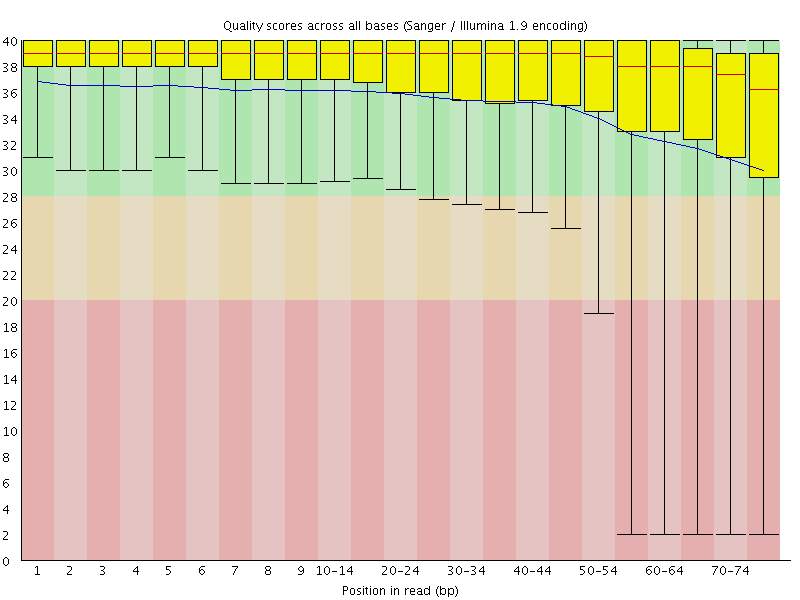

Supplement: Additional file 3: Figure S2. — Visualization for RNA transcript quality control and comparison of per base quality score Q. The images are taken before (A) and after (B) quality trimming procedure (removes reads with Q ≤ 20) to estimate the effect of trimming. The quality score Q is plotted to the read position by using the FastQC package in Galaxy (http://www.bioinformatics.babraham.ac.uk/projects/fastqc/). The color indicates the quality of the read: "red" low quality, "orange" median quality, "green" good quality. Red line expresses the mean of the measured values (yellow boxes are inter-quartile range) and the blue line represents the mean quality. (ZIP 81 kb) [file 12859_2015_873_MOESM3_ESM.zip › figure s2/FigureS2A.tif]

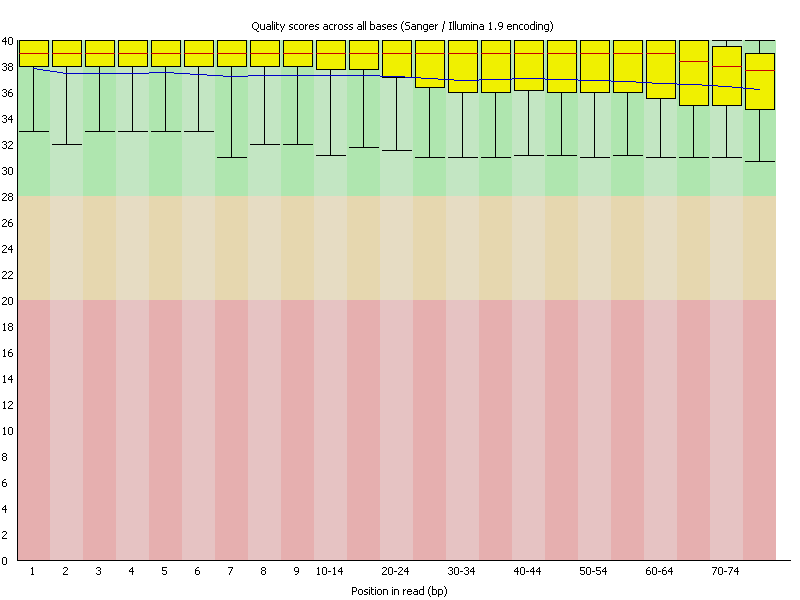

Supplement: Additional file 3: Figure S2. — Visualization for RNA transcript quality control and comparison of per base quality score Q. The images are taken before (A) and after (B) quality trimming procedure (removes reads with Q ≤ 20) to estimate the effect of trimming. The quality score Q is plotted to the read position by using the FastQC package in Galaxy (http://www.bioinformatics.babraham.ac.uk/projects/fastqc/). The color indicates the quality of the read: "red" low quality, "orange" median quality, "green" good quality. Red line expresses the mean of the measured values (yellow boxes are inter-quartile range) and the blue line represents the mean quality. (ZIP 81 kb) [file 12859_2015_873_MOESM3_ESM.zip › figure s2/FigureS2B.tif]

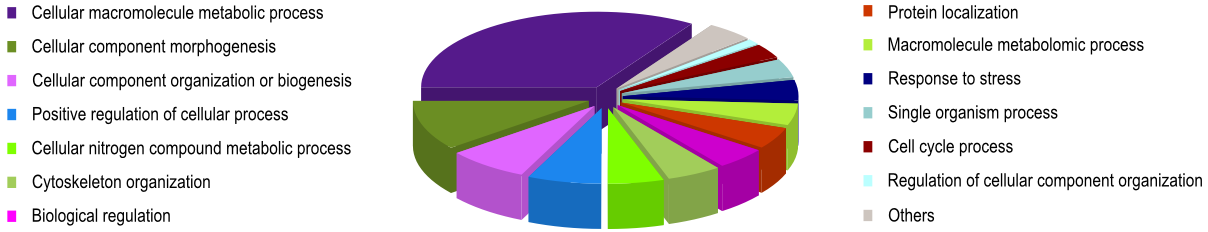

Supplement: Additional file 6: Table S3. — Exemplarily we show a result of a miRNA target prediction analysis of TRAPLINE. (TIF 84 kb) [file 12859_2015_873_MOESM6_ESM.tif]

## Slide 1
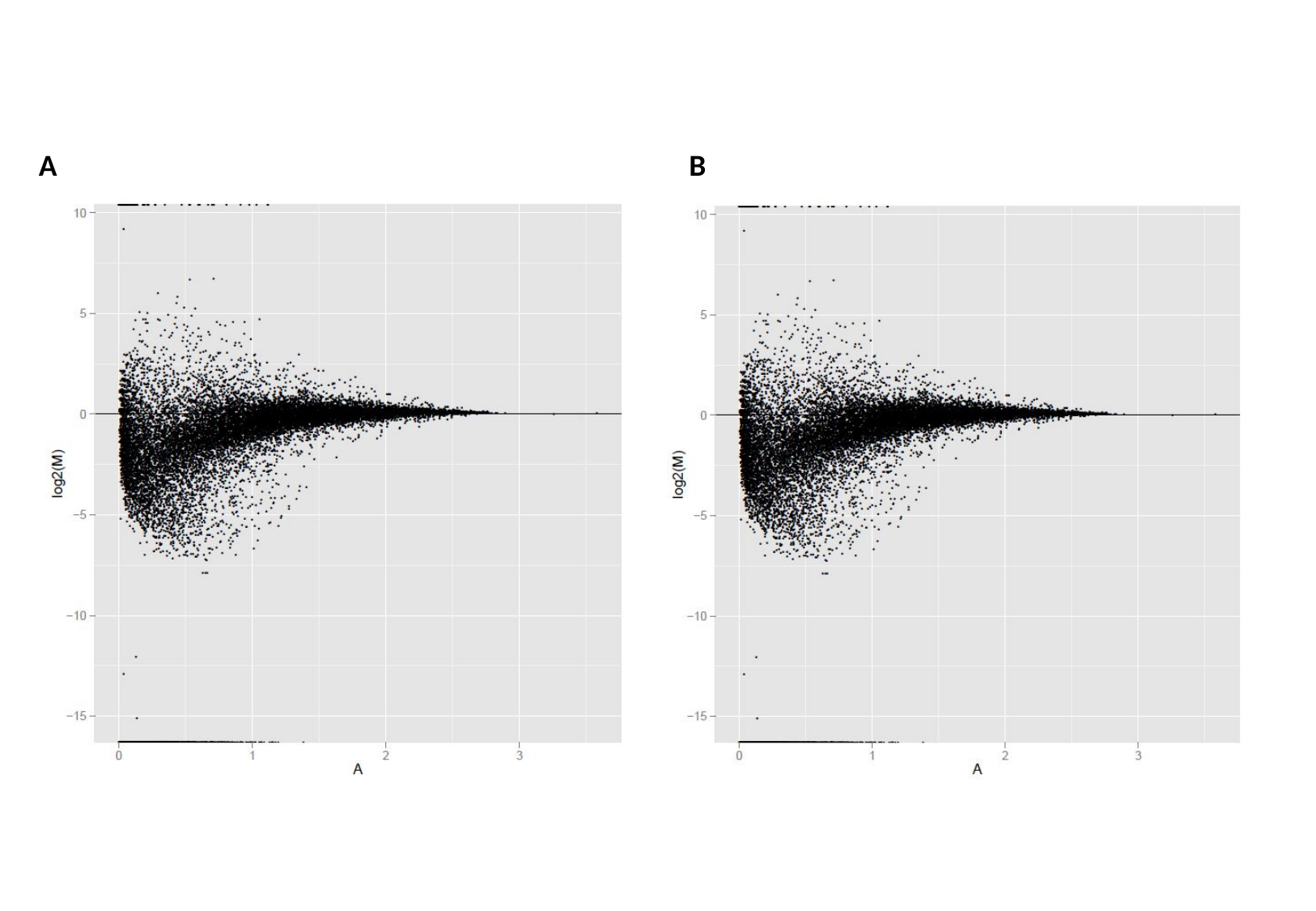

A
B

Supplement: Additional file 10: Figure S4. — A comparison of experiments without (A) and with (B) bias correction performed with the help of Cufflinks2. The dots represent the dependency of the log ratio of two FPKM values (M) and their mean average (A). The MA plot is a common method to investigate the biases of datasets [53]. (PPTX 236 kb) [file 12859_2015_873_MOESM10_ESM.pptx]
