# Supplementary material for: TRAPLINE: a standardized and automated pipeline for RNA sequencing data analysis, evaluation and annotation
Source: BMC Bioinformatics. 2016 Jan 6;17:21. doi: 10.1186/s12859-015-0873-9 (PMC4702420; doi:10.1186/s12859-015-0873-9)
Supplement: Additional file 4: Table S1. — Performance comparison of TRAPLINE vs other tools. (DOC 28 kb) [file 12859_2015_873_MOESM4_ESM.doc]

Table S1: Performance comparison of TRAPLINE *vs* other tools.

| metric | TRAPLINE | BWA | Bowtie | RSeQC | edgeR | SAMseq |
| --- | --- | --- | --- | --- | --- | --- |
| mapped reads in % | ~80 (TopHat2) | ~72 | ~71 |  |  |  |
| quality control - discarded sequences in million | ~2.9  (FASTX TK) |  |  | ~2.9 |  |  |
| # diff. expr. genes | 550 (Cuffdiff2) |  |  |  | 370 | 475 |
