# Supplementary material for: TRAPLINE: a standardized and automated pipeline for RNA sequencing data analysis, evaluation and annotation
Source: BMC Bioinformatics. 2016 Jan 6;17:21. doi: 10.1186/s12859-015-0873-9 (PMC4702420; doi:10.1186/s12859-015-0873-9)
Supplement: Additional file 5: Table S2. — Example for DAVID functional gene annotation clustering of significantly differentially expressed genes from aCaBs and EBs. (DOC 48 kb) [file 12859_2015_873_MOESM5_ESM.doc]

Table S1. Example for DAVID functional gene annotation clustering of significantly differentially expressed genes from aCaBs and EBs.

| **Annotation Cluster 1** | **Enrichment Score: 7,72** |  |  |  |
| --- | --- | --- | --- | --- |
| **Category** | **Term** | **Count** | **PValue** | **FDR** |
| GOTERM_CC_FAT | GO:0043228~non-membrane-bounded organelle | 34 | 7,17E-03 | 1,06E-08 |
| GOTERM_CC_FAT | GO:0043232~intracellular non-membrane-bounded organelle | 34 | 7,17E-03 | 1,06E-08 |
| SP_PIR_KEYWORDS | cytoskeleton | 13 | 2,72E-07 | 4,00E-10 |
| GOTERM_CC_FAT | GO:0005856~cytoskeleton | 20 | 2,10E-08 | 3,09E-11 |
| GOTERM_CC_FAT | GO:0015630~microtubule cytoskeleton | 8 | 2,25E-10 | 3,30E-02 |
| GOTERM_CC_FAT | GO:0044430~cytoskeletal part | 13 | 6,98E-11 | 10,22E-13 |
| **Annotation Cluster 2** | **Enrichment Score: 7,19** |  |  |  |
| **Category** | **Term** | **Count** | **PValue** | **FDR** |
| GOTERM_MF_FAT | GO:0008092~cytoskeletal protein binding | 38 | 5,50E-05 | 8,92E-08 |
| GOTERM_MF_FAT | GO:0003779~actin binding | 30 | 6,99E-07 | 1,13E-12 |
| GOTERM_CC_FAT | GO:0015629~actin cytoskeleton | 23 | 6,30E-08 | 9,27E-11 |
| SP_PIR_KEYWORDS | actin-binding | 29 | 6,90E-08 | 10,14E-04 |
| **Annotation Cluster 3** | **Enrichment Score: 6,44** |  |  |  |
| **Category** | **Term** | **Count** | **PValue** | **FDR** |
| GOTERM_CC_FAT | GO:0043292~contractile fiber | 33 | 1,09E-09 | 1,60E-12 |
| GOTERM_CC_FAT | GO:0044449~contractile fiber part | 31 | 1,12E-08 | 1,65E-12 |
| GOTERM_CC_FAT | GO:0030016~myofibril | 22 | 1,28E-08 | 1,89E-11 |
| GOTERM_CC_FAT | GO:0030017~sarcomere | 19 | 2,72E-08 | 4,00E-12 |
| GOTERM_CC_FAT | GO:0031674~I band | 12 | 8,06E-08 | 1,19E-03 |
| GOTERM_CC_FAT | GO:0030018~Z disc | 9 | 6,06E-09 | 8,914E-03 |
