# Supplementary material for: TRAPLINE: a standardized and automated pipeline for RNA sequencing data analysis, evaluation and annotation
Source: BMC Bioinformatics. 2016 Jan 6;17:21. doi: 10.1186/s12859-015-0873-9 (PMC4702420; doi:10.1186/s12859-015-0873-9)
Supplement: Additional file 7: Figure S3. — Pie chart illustrating enriched biological processes of upregulated genes in the aCaB derived cardiomyocytes. The chart presents the enriched GO superclasses. (DOC 26 kb) [file 12859_2015_873_MOESM7_ESM.doc]

Table S1. Exemplarily we show a result of a miRNA target prediction analysis of TRAPLINE.

| **MiRNA name** | **Gene ID** | **Gene symbol** | **Genome coordinates** | **align score** | **GO term** |
| --- | --- | --- | --- | --- | --- |
| Mir369-3p | 98660 | Atp1a2 | mm9:1:174204547-174204565:- | 152 | GO:0045822 |
