# Supplementary material for: TRAPLINE: a standardized and automated pipeline for RNA sequencing data analysis, evaluation and annotation
Source: BMC Bioinformatics. 2016 Jan 6;17:21. doi: 10.1186/s12859-015-0873-9 (PMC4702420; doi:10.1186/s12859-015-0873-9)
Supplement: Additional file 8: Table S4. — Exemplarily results of protein-protein interaction prediction, splice variants and multi promoter regions. (DOC 37 kb) [file 12859_2015_873_MOESM8_ESM.doc]

Table S1. Exemplarily results of protein-protein interaction prediction, splice variants and multi promoter regions.

| **Protein-protein interactions** | | | |  | |  | |  | |  | |  |
| --- | --- | --- | --- | --- | --- | --- | --- | --- | --- | --- | --- | --- |
| **Gene 1** | **Gene 2** | | **Capture Method** | | | | | **Interaction type** | | **Reference** | | **Pubmed ID** |
| Atp1a2 | Slc8a1 | | Affinity Capture-Western | | | | | physical | | Dostanic I (2004) | | 15485817 |
| Mef2c | Nkx2-5 | | Affinity Capture-Western | | | | | physical | | Vincentz JW (2008) | | 19035347 |
| Tnni3 | Tnnt2 | | Co-crystal Structure | | | | | physical | | Heller WT (2002) | | 12501194 |
| **Splice variants** |  | |  | | | | |  | |  | |  |
| **Gene** | **Location** | | | | | | | **p-value** | | **q-value** | | **significant** |
| Tnnt2 | chr1:137732962-137748838 | | | | | | | 5E-05 | | 1E-03 | | yes |
| **Multi promoter regions** | |  | | |  | |  | |  | |  | |
| **Gene** | **TSS** | | **Location** | | | | | **p-value** | | **q-value** | | **significant** |
| Tnnt2 | 447 | | chr1:137732962-137748838 | | | | | 5E-05 | | 2E-04 | | yes |
| Tcf19 | 9977 | | chr17:35649679-35653769 | | | | | 2E-04 | | 5E-03 | | yes |
