# Supplementary material for: TRAPLINE: a standardized and automated pipeline for RNA sequencing data analysis, evaluation and annotation
Source: BMC Bioinformatics. 2016 Jan 6;17:21. doi: 10.1186/s12859-015-0873-9 (PMC4702420; doi:10.1186/s12859-015-0873-9)
Supplement: Additional file 9: Table S5. — Benchmarking results of TRAPLINE performed on a public Galaxy server and on a local desktop PC (based on computing speed). (DOC 31 kb) [file 12859_2015_873_MOESM9_ESM.doc]

Table S1. Benchmarking results of TRAPLINE performed on a public Galaxy server and on a local desktop PC (based on computing speed).

| **Datasets** |  | **Computing speed desktop PC** | **Computing speed TRAPLINE** |
| --- | --- | --- | --- |
| Our use case of cardiac cell types |  | ~24 h | ~10 h |
| Randomly selected use case from SRA  (PRJNA292442) [50] |  | ~32 h | ~15 h |
